# Melatonin and erastin emerge synergistic anti-tumor effects on oral squamous cell carcinoma by inducing apoptosis, ferroptosis, and inhibiting autophagy through promoting ROS

**DOI:** 10.1186/s11658-023-00449-6

**Published:** 2023-05-02

**Authors:** Leilei Wang, Chuan Wang, Xuan Li, Zhuoying Tao, Wangyong Zhu, Yuxiong Su, Wing Shan Choi

**Affiliations:** 1 Division of Oral and Maxillofacial Surgery, Faculty of Dentistry, The University of Hong Kong, 34 Hospital Road, Hong Kong SAR, China; 2 The State Key Laboratory Breeding Base of Basic Science of Stomatology (Hubei-MOST KLOS) and Key Laboratory of Oral Biomedicine Ministry of Education (KLOBME), School and Hospital of Stomatology, Wuhan University, Wuhan, China; 3 Department of Periodontology, School and Hospital of Stomatology, Wuhan University, Wuhan, China; 4 Division of Periodontology and Implant Dentistry, Faculty of Dentistry, The University of Hong Kong, Hong Kong SAR, China; 5 Department of Dental Surgery, The University of Hong Kong-Shenzhen Hospital, Shenzhen, Guangdong China

**Keywords:** Reactive oxygen species (ROS), Oral cancer, Melatonin, Erastin, Apoptosis, Autophagy, Ferroptosis

## Abstract

**Background:**

Oral squamous cell carcinomas are one of the most common cancers worldwide with aggressive behavior and poor prognosis. Reactive oxygen species (ROS) are associated with cancer and cause various types of regulated cell death (RCD). Inducing the RCD pathway by modulating ROS levels is imperative to conquer cancers. The aim of this study is to investigate the synergistic anticancer effects of melatonin and erastin on ROS modulation and subsequent RCD induction.

**Methods:**

Human tongue squamous cell carcinoma cell lines (SCC-15 cells) were treated with melatonin, erastin, or their combination. Cell viability, ROS levels, autophagy, apoptosis, and ferroptosis levels were tested according to the results of the PCR array, which were verified with/without the induction and inhibition of ROS by H_2_O_2_ and N-acetyl-L-cysteine, respectively. In addition, a mouse-based subcutaneous oral cancer xenograft model was constructed to identify the effects of melatonin, erastin, and their combination on the autophagy, apoptosis, and ferroptosis levels in isolated tumor tissues.

**Results:**

ROS levels were increased by the administration of melatonin at high concentrations (mM), and the combination of melatonin with erastin enhanced the levels of malonic dialdehyde, ROS, and lipid ROS, and reduced the levels of glutamate and glutathione. SQSTM1/p62, LC3A/B, cleaved caspase-3, and PARP1 protein levels in SCC-15 cells were also increased by melatonin plus erastin treatment, which further increased as ROS accumulated, and decreased as ROS levels were suppressed. Combined treatment of melatonin and erastin markedly reduced the tumor size in vivo, demonstrated no obvious systemic side effects, and significantly enhanced the apoptosis and ferroptosis levels in the tumor tissues, in parallel with decreased autophagy levels.

**Conclusions:**

Melatonin combined with erastin exhibits synergistic anticancer effects without adverse reactions. Herein, this combination might become a promising alternative strategy for oral cancer treatment.

**Graphical Abstract:**

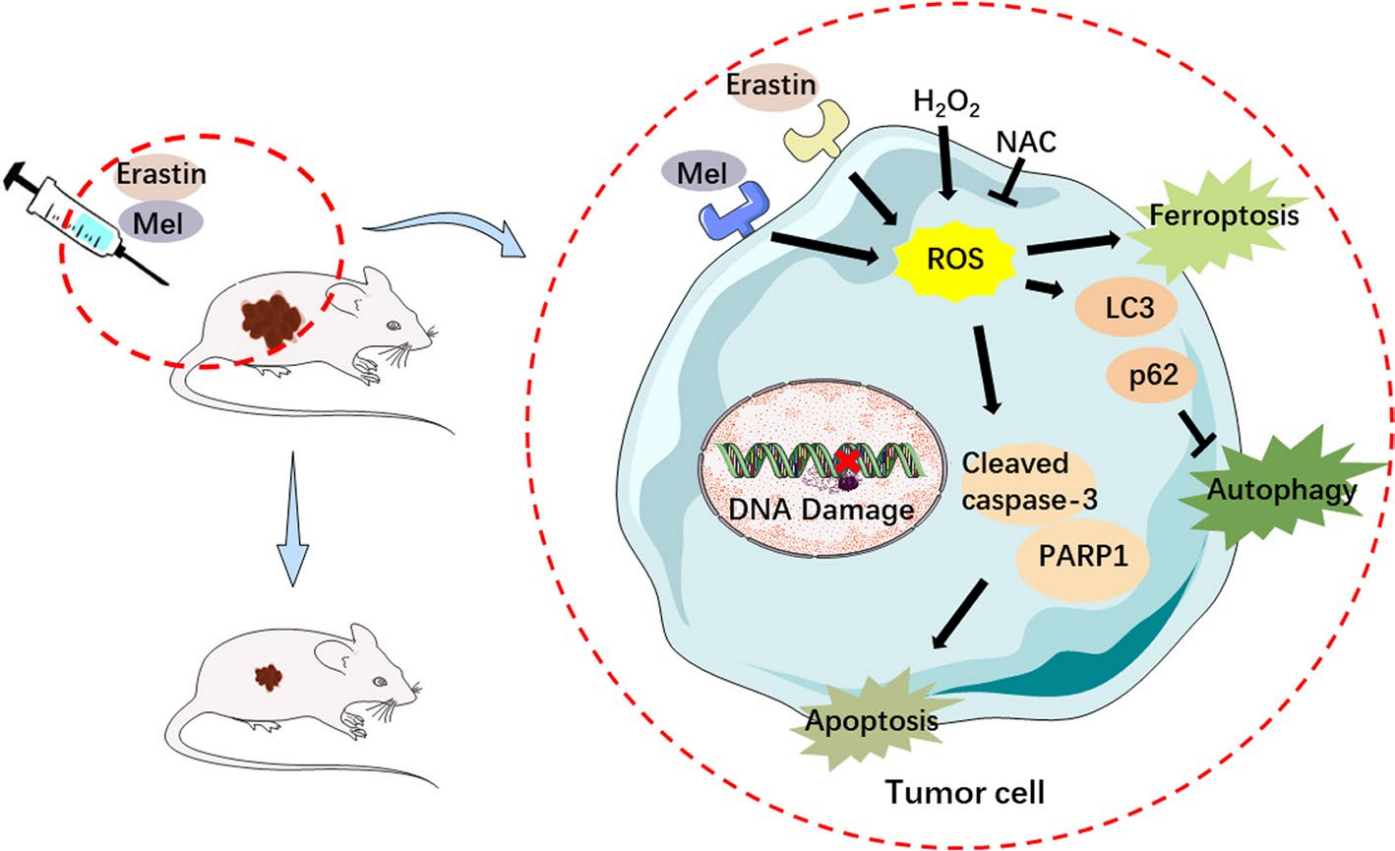

**Supplementary Information:**

The online version contains supplementary material available at 10.1186/s11658-023-00449-6.

## Background

Oral cancers are a group of cancers that have high morbidity and mortality and affect nearly 600,000 people worldwide each year [1]. To date, surgery followed by adjuvant chemo- or radiotherapy is still the most widely adopted treatment modality for oral squamous cell carcinomas (OSCCs). However, the long-term survival rate of OSCCs is poor, with a 10-year survival rate of only 20% [2]. Therefore, more effective drugs and treatment strategies are warranted to improve the prognosis of patients with OSCC.

Increasing evidence has demonstrated that alterations in the redox balance affects tumor progression and resistance to treatment [3]. Redox homeostasis depends on the balance of oxidant production and elimination over time. Reactive oxygen species (ROS) are unstable molecules derived from oxygen that have unpaired electrons. They are generated, transformed, and eliminated in many cellular processes, including metabolism, proliferation, differentiation, and immune system regulation [4]. Low levels of ROS have advantageous effects on cell growth, while abundant ROS levels lead to DNA, lipid, and protein damage and even cell death. Since cancer cells exhibit enhanced metabolism and intense proliferation, they produce higher ROS levels than those produced by normal cells. To maintain redox homeostasis, tumor cells strategically modulate multiple antioxidant enzymes and make extensive use of their metabolic pathways to provide an adequate supply of antioxidant molecules, such as glutathione (GSH) [5]. The GSH system is one of the major cellular antioxidant systems [6], and its depletion triggers multiple forms of programmed cell death in cancer cells, such as apoptosis, necroptosis, autophagy, and ferroptosis [7]. Therefore, treating cancer cells with ROS-inducing or antioxidant-inhibiting strategies could be an effective tool in anticancer therapy.

Regulated cell death (RCD) plays an important role in cancer therapy. There are many different types of RCD, such as apoptosis, autophagy, and ferroptosis [8]. Most conventional cancer chemotherapies, such as cisplatin, primarily induce apoptosis [9]. However, cancer cells may develop resistance to chemotherapy and avoid the induction of apoptosis [10]. Thus, an increasing number of studies have developed combination therapy to overcome this problem. Inducing different types of RCD could be a promising strategy.

Ferroptosis, first coined in 2012 by Dixon et al. [11], is a new form of iron-dependent oxidative cell death modulated by specific genetic or pharmacological manipulations. It is morphologically, biochemically, and genetically distinct from apoptosis, autophagy, and necrosis. Ferroptosis can be described as a process of lipid peroxidation accumulation [12]: excessive substrate for peroxidation, a lack of the redox-active iron to drive the peroxidation process, and the failure of lipid peroxidation elimination by the repair system. Inducing ferroptosis in cancer cells is a novel and feasible mechanism for cancer treatment [13]. Numerous studies have shown that ferroptosis stimulators play an important role in suppressing tumor growth and killing tumor cells, including glioblastoma cells, non-small cell lung cancer cells, and head and neck cancer cells [14–16]. Erastin, a classical ferroptosis stimulator [17], can inhibit the function of cystine–glutamate antiporter system Xc^−^ [18]. Thus, cells treated with erastin fail to synthesize the antioxidant glutathione, which eventually leads to excessive lipid peroxidation and cell ferroptosis. The role of ferroptosis in tumor cells is not fully understood due to the vague underlying mechanism and targeting pathway, and erastin is a representative drug to study the mechanism of ferroptosis.

Melatonin (N-acetyl-5-methoxytryptamine) is an endogenous hormone that possesses various biological functions, including antioxidant, antiinflammatory, and immune system regulatory properties [19]. It has been proven to be a natural oncostatic agent that can inhibit tumor growth by inducing apoptosis in cancer cells [20]. It also has inhibitory and stimulatory effects on cancer cell autophagy [21]. Several studies have illuminated the inhibitory effect of melatonin on ferroptosis in osteoporosis [22] and acute kidney injury [23], while the effect of melatonin on cancer cell ferroptosis has not been reported in the literature.

In the present study, we investigated the anticancer efficacy and side effects of melatonin combined with erastin in vitro and in vivo. Moreover, we also explored the possible regulators that modulate ferroptosis, autophagy, and apoptosis. These results will pave the way for novel treatment strategies to increase the therapeutic efficacy of oral cancer treatment.

## Methods

### Cell line and cell culture

The human tongue squamous cell carcinoma cell line SCC-15 was purchased from American Type Culture Collection (ATCC, Manassas, VA, USA) and maintained in DMEM: F12 [Dulbecco’s modified Eagle’s medium: Nutrient Mixture F-12 (Gibco, USA)], supplied with 10% FBS, 100 U/mL penicillin‒streptomycin, and 400 ng/mL hydrocortisone (Sigma‒Aldrich, USA).

### Iron assay

Cell lysates and mouse serum were collected. Intracellular ferrous iron levels were measured using an iron assay kit (Abcam, ab83366) according to the manual instructions.

### Intracellular glutamate assay

The relative levels of intracellular glutamate were measured using the Glutamate Assay Kit (Abcam, #ab83389) according to the manufacturer’s instructions. Glutamate levels were normalized to the total cell number (measured by CCK-8 kit) and were then illustrated as a percentage of the control group.

### Immunohistochemistry (IHC) analysis

Paraffin-embedded tissues were sectioned forIHC analysis. Samples were fixed in 10% formalin and embedded in paraffin wax. Five-millimeter sections were cut from the paraffin blocks and stained with cleaved caspase-3 (CST, Danvers, MA, USA, 9661, 1:200), PARP1 monoclonal antibody (Thermo Fisher Scientific Inc., Grand Island, NY, USA, 436400, 1:20), SQSTM1/p62 (CST, Danvers, MA, USA, 23214, 1:200), or LC3A/B (CST, Danvers, MA, USA, 12741, 1:400) at 4 °C overnight. All sections were covered with neutral balsam, viewed with an Olympus microscope, and analyzed with ImageJ software. The statistical analyses for each target were the average score of all fields of view.

### Western blot analysis

The total proteins were first separated by 10% sodium dodecyl sulfate‒polyacrylamide gel electrophoresis (SDS‒PAGE) and then electrophoretically transferred onto supported nitrocellulose membranes. Next, they were blocked with 5% skimmed milk in PBST for 2 h. After incubation with primary antibodies overnight at 4 °C, the membranes were incubated with the secondary antibodies for 2 h at room temperature. The blots on the membranes were visualized by ECL hypersensitive luminescence (Thermo Scientific, Waltham, MA, USA) and detected by ChemiDoc Touch Imaging System with Image Lab Touch Software (Bio-Rad, Hercules, USA).

### Cell viability assay

The effect of melatonin and erastin on cell viability was assessed by a Vybrant MTT cell proliferation assay kit (Thermo Fisher Scientific Inc., Grand Island, NY, USA, V-13154). The cells were seeded into 96-well plates at a density of 5000 cells/well. Then the cells were treated with 0.5 mM melatonin, 0.5 μM erastin, 0.5 mM melatonin + 0.5 μM erastin, 2 mM melatonin, 5 μM erastin, and 2 mM melatonin + 5 μM erastin for 24 h and 48 h. MTT solution at a concentration of 5 mg/mL was added to each well. After 2.5 h of incubation, the reaction was stopped by adding DMSO. The absorbance of each well at 540 nm was measured by a SpectraMax M2 microplate reader (Molecular Device, San Jose, USA).

### MDA assay

A thiobarbituric acid reactive substances (TBARS) assay kit (Cayman Chemical, Ann Arbor, MI, USA, 10009055) was used to monitor lipid peroxidation in cells and mouse serum. In the in vitro study, 2 × 10^7^ cells were collected and homogenized on ice, and then diluted to the relevant concentrations for further procedures according to the product instructions. Simultaneously, the concentration of protein lysates was determined using the BCA Protein Assay to normalize the MDA level.

### Glutathione quantification

The cells were seeded into a white, clear bottom 96-well plate (2000 cells per well) overnight. Then, the cells were treated with melatonin, erastin, and melatonin + erastin for 48 h. GSH levels were measured using the GSHGlo Glutathione Assay kit (Promega, Madison, WI, USA, V6912), following the manufacturer’s instructions. Luminescence was measured using an LMax Microplate Reader (Molecular Devices). A standard curve for GSH concentration was generated along with samples and used for calculation. In addition, cell viability in another set of wells was measured using the MTT assay. The GSH concentration in each group was normalized to cell viability.

### Lipid peroxidation assessed by BODIPY™ 581/591 C11 (lipid peroxidation sensor)

The cells (2000 cells per well) were seeded in 96-well plates. C11-BODIPY dye (Thermo Fisher Scientific Inc., Grand Island, NY, USA, #D3861) was used to detect lipid peroxidation in cells according to the manufacturer’s instructions. The fluorescence intensity was evaluated using fluorescence microscopy.

### DCFDA/H2DCFDA—cellular ROS assay

The cells (5000 cells per well) were seeded in a dark, clear bottom 96-well microplate overnight. Then, the cells were treated with 0.5 mM melatonin, 0.5 μM erastin, 0.5 mM melatonin + 0.5 μM erastin, 2 mM melatonin, 5 μM erastin, and 2 mM melatonin + 5 μM erastin for 24 h. After removing the media, the cells were stained using the DCFDA/H2DCFDA-Cellular ROS Assay Kit (Abcam, Cambridge, UK, ab113851), according to the manufacturer’s instructions. The plate was read by a fluorescence plate reader at Ex/Em = 485/535 nm at the end point. In addition, cell viability in another set of wells was measured using the MTT assay. The ROS concentration in each group was normalized to cell viability.

### RT^2^ profiler PCR arrays

cDNA was synthesized from 1 μg of extracted RNA using the PrimeScript RT Reagent Kit (TaKaRa, Shiga, Japan). Then, PCR component mixtures were prepared in a 5 mL tube as follows: 1350 µL 2 × RT^2^ SYBR Green Mastermix, 650 µL cDNA, and 2100 µL RNase-free water. After adding 25 µL of PCR component mix to each well of the RT^2^ Profiler PCR Array (QIAGEN, Hilden, Düsseldorf, Germany, 330231), qPCR amplifications were performed on a StepOnePlus, according to the setup instructions www.qiagen.com/shop/pcr/primer-sets/rt2-profiler-pcr-arrays/#resource. The results (Additional file 1: Figure S1A-B) were analyzed via the tools from the website: https://geneglobe.qiagen.com/us/analyze.

### Flow cytometry

Cell apoptosis was evaluated using a Dead Cell Apoptosis Kit with Annexin V Alexa Fluor 488 and PI (Thermo Fisher Scientific, Waltham, MA, USA, V13245), according to the manufacturer’s protocol. In brief, cells were collected and washed three times in cold phosphate-buffered saline (PBS). After resuspension in 1 × annexin-binding buffer, 5 µL Alexa Fluor 488 annexin V and 1 µL of 100 µg/mL PI working solution were added to each 100 µL of cell suspension. Fifteen minutes later, 400 µL 1 × annexin-binding buffer was added to the cell suspension. Cells were analyzed through flow cytometry immediately after staining (BD FACS CantoII Analyzer, BD, Franklin Lake, NJ, USA).

### Animal experiments

All experimental procedures were approved by the Committee on the Use of Live Animals in Teaching and Research (CULATR, HKU). BALB/c nude mice (4–5 weeks old, male/female) were maintained in a specific pathogen-free facility (Lab Animal Unit, LAU, HKU). A total of 1 × 10^6^ SCC-15 cells suspended in 100 μL PBS were injected subcutaneously into the backs of the mice. One week after injection, 24 mice were randomly divided into four groups: (1) blank control, (2) intraperitoneal injection with 200 μL of a solution of melatonin (100 mg/kg), (3) intraperitoneal injection with 200 μL of a solution of erastin (30 mg/kg), and (4) intraperitoneal injection with 200 μL of a solution of melatonin (100 mg/kg) and erastin (30 mg/kg). All treatments were given every 2 days for seven cycles. The mice were housed in a 12 h light/12 h dark cycle with food and water supplied. Tumor growth and body weight were monitored. Tumor length and width were measured using calipers every 2 days. Tumor volume was calculated as follows: tumor volume = 0.5a × b^2^ (a: length, b: width). After sacrificing the mice, the volume and wet weight of the tumors were measured, and blood was collected from the heart.

### Statistical analysis

The differences between two groups were calculated by Student’s *t* test, and the differences among groups were calculated by one-way ANOVA. All experiments were repeated independently at least three times. The data were analyzed using GraphPad Prism 7.0 and are presented as the mean ± standard deviation (SD); *p* < *0.05* was considered statistically significant.

## Results

### Melatonin combined with erastin decreased SCC-15 cells viability and increased the cellular ROS level

**Fig. 1:**
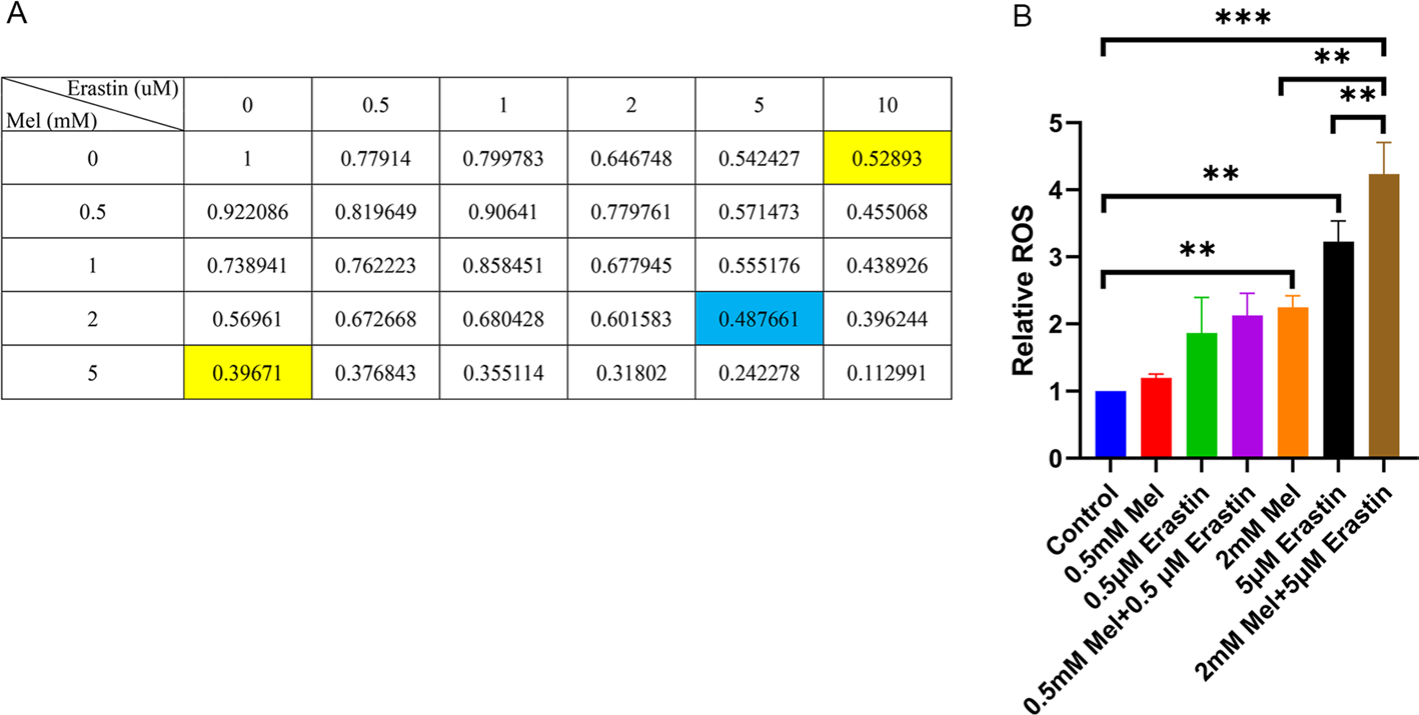
Effects of melatonin and erastin on SCC-15 cells viability and ROS expression. SCC-15 cells were treated with erastin (0, 0.5, 1, 2, 5, 10 μM), melatonin (0, 0.5, 1, 2, 5 mM), and their combination for 48 h with MTT assay conducted (**A**), and for 24 h with relative ROS tested (**B**) (*p < 0.05, **p < 0.01, ***p < 0.001)

To test whether the combined use of melatonin and erastin has a greater anticancer efficiency against SCC-15 cells, MTT assays were performed and the half-maximal inhibitory concentration (IC50) was calculated. Different combinations of melatonin (0 mM, 0.5 mM, 1 mM, 2 mM, and 5 mM) and erastin (0 μM, 0.5 μM, 1 μM, 2 μM, and 5 μM) were added to SCC-15 cells for 48 h. The results showed that the IC50 values of melatonin and erastin when used separately were 5 mM and more than 10 μM, respectively (Fig. 1A, marked as yellow). Notably, it was highly achievable to decrease the viability of SCC-15 cells with lower dosages of each drug when used in combination (Fig. 1A, marked as blue).

The cellular ROS levels in SCC-15 cells were tested and compared between high and low concentrations of melatonin and erastin (Fig. 1B). Notably, 2 mM melatonin significantly increased the cellular ROS level, while 0.2 mM melatonin did not yield any significant effect. In addition, higher ROS levels were produced with the combined use of 2 mM melatonin and 5 μM erastin.

### Melatonin combined with erastin decreased cell autophagy and increased apoptotic and ferroptotic death

**Fig. 2:**
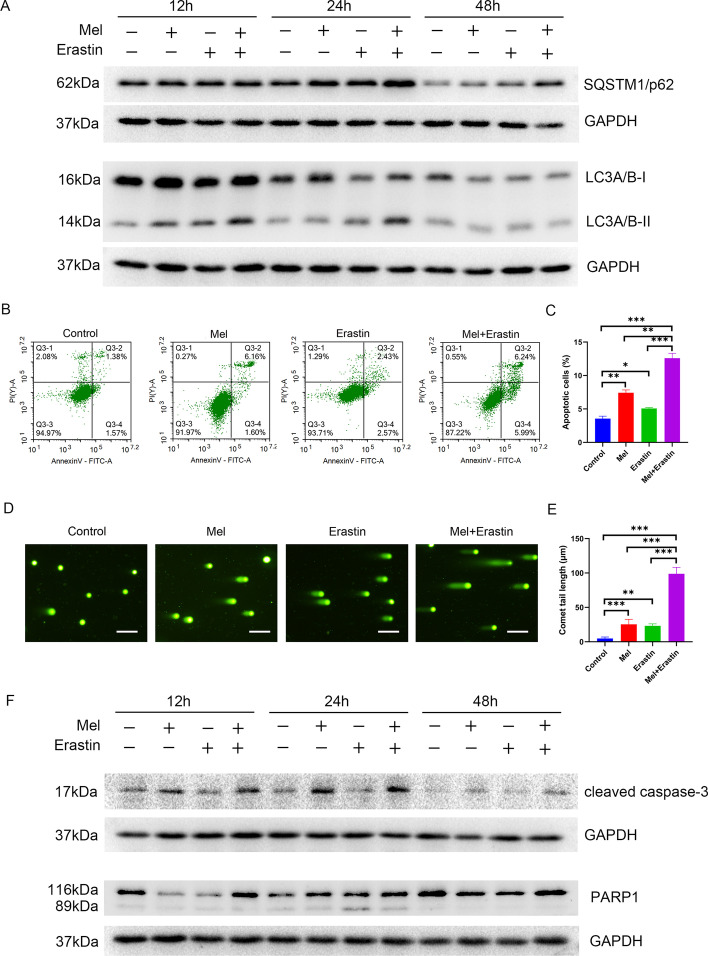
Effects of melatonin and erastin on autophagy and apoptosis of SCC-15 cells. SCC-15 cells were treated with melatonin (2 mM), erastin (5 μM), and their combination for 12 h, 24 h, and 48 h. The protein expression levels of SQSTM1/p62 and LC3A/B were measured (**A**). Flow cytometry (**B**) and comet assay (**D**) were conducted (Additional file 1: Figure S2).** C** and** E** Quantitative data from three independent experiments are shown in the right panels, respectively. The protein expression levels of cleaved caspase-3 and PARP1 were tested (**E**). *ns* not significant, *p < 0.05, **p < 0.01, ***p < 0.001. Scale bars: 100 nm

After treatment with 2 mM melatonin and 5 μM erastin for 12 h, 24 h, and 48 h, the expression levels of SQSTM1/p62 and LC3A/B in SCC-15 cells were significantly increased, and the cells treated with melatonin + erastin exerted the highest expression levels among the four groups (Fig. 2A). Thus, melatonin combined with erastin interrupted the autophagy process in SCC-15 cells.

Moreover, as verified by flow cytometry (Fig. 2B), more apoptotic SCC-15 cells were found in the melatonin + erastin-treated group than in the melatonin- or erastin-treated group (Fig. 2C). In addition, apoptotic DNA fragmentation in the cells was assessed through a comet assay (Fig. 2D), and the longest comet tail was found in the melatonin + erastin-treated group, suggesting that SCC-15 cells treated with melatonin + erastin resulted in the greatest number of DNA breaks (Fig. 2E). In addition, the expression levels of PARP1 and cleaved caspase-3 were significantly increased in the melatonin- and melatonin + erastin-treated groups. The cells treated with melatonin + erastin exhibited the highest expression levels among the four groups at 24 h (Fig. 2F). Interestingly, the expression levels of PARP1 and cleaved caspase-3 in the erastin-treated group showed no difference from those in the control group at both 24 h and 48 h. Therefore, apoptosis in SCC-15 cells was stimulated by the combination of melatonin and erastin.

**Fig. 3:**
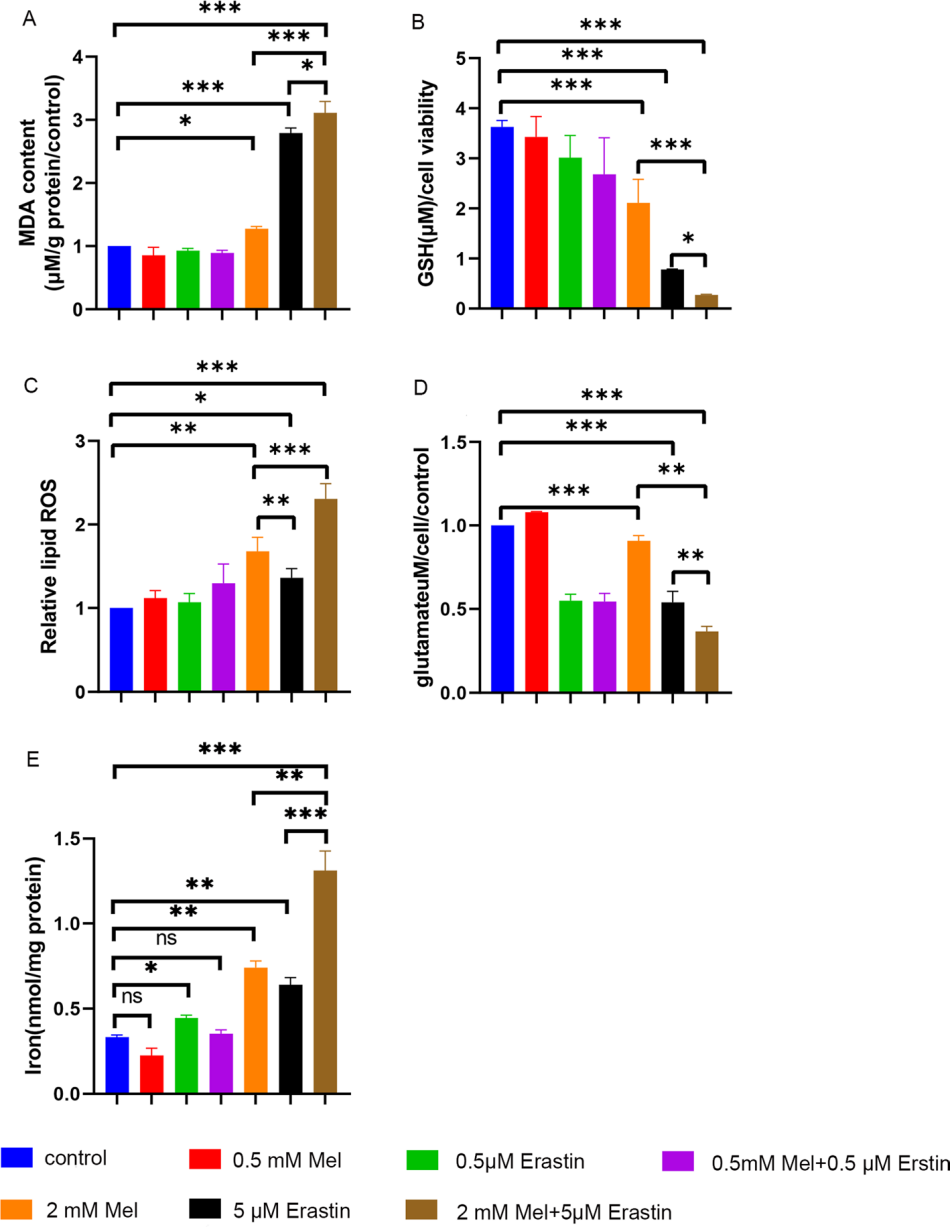
Effects of melatonin and erastin on ferroptosis of SCC-15 cells. SCC-15 cells were treated with erastin (0, 0.5, 5 μM), melatonin (0, 0.5, 2 mM), and their combination for 24 h. The MDA content (**A**), GSH concentration (**B**), relative lipid ROS (**C**), glutamate concentration (**D**), and iron level (**E**) were tested. *ns* not significant, *p < 0.05, **p < 0.01, ***p < 0.001

To investigate the change in the ferroptosis level in SCC-15 cells by the combined oncostatic treatment, SCC-15 cells were treated with different concentrations of melatonin and erastin. The results showed that a high concentration of melatonin significantly increased the MDA level (Fig. 3A), relative lipid ROS level (Fig. 3C), and iron level (Fig. 3E), in parallel with the decreased GSH (Fig. 3B) and glutamate levels (Fig. 3D). Notably, compared with their single usage, the combination of melatonin and erastin remarkably increased the levels of these metrics (Fig. 3A–E). However, with low concentrations of melatonin and erastin, these changes varied greatly and did not affect ferroptosis levels in SCC-15 cells.

### The inhibition of autophagy, and enhancement of apoptosis and ferroptosis by melatonin and erastin were regulated by ROS levels

**Fig. 4:**
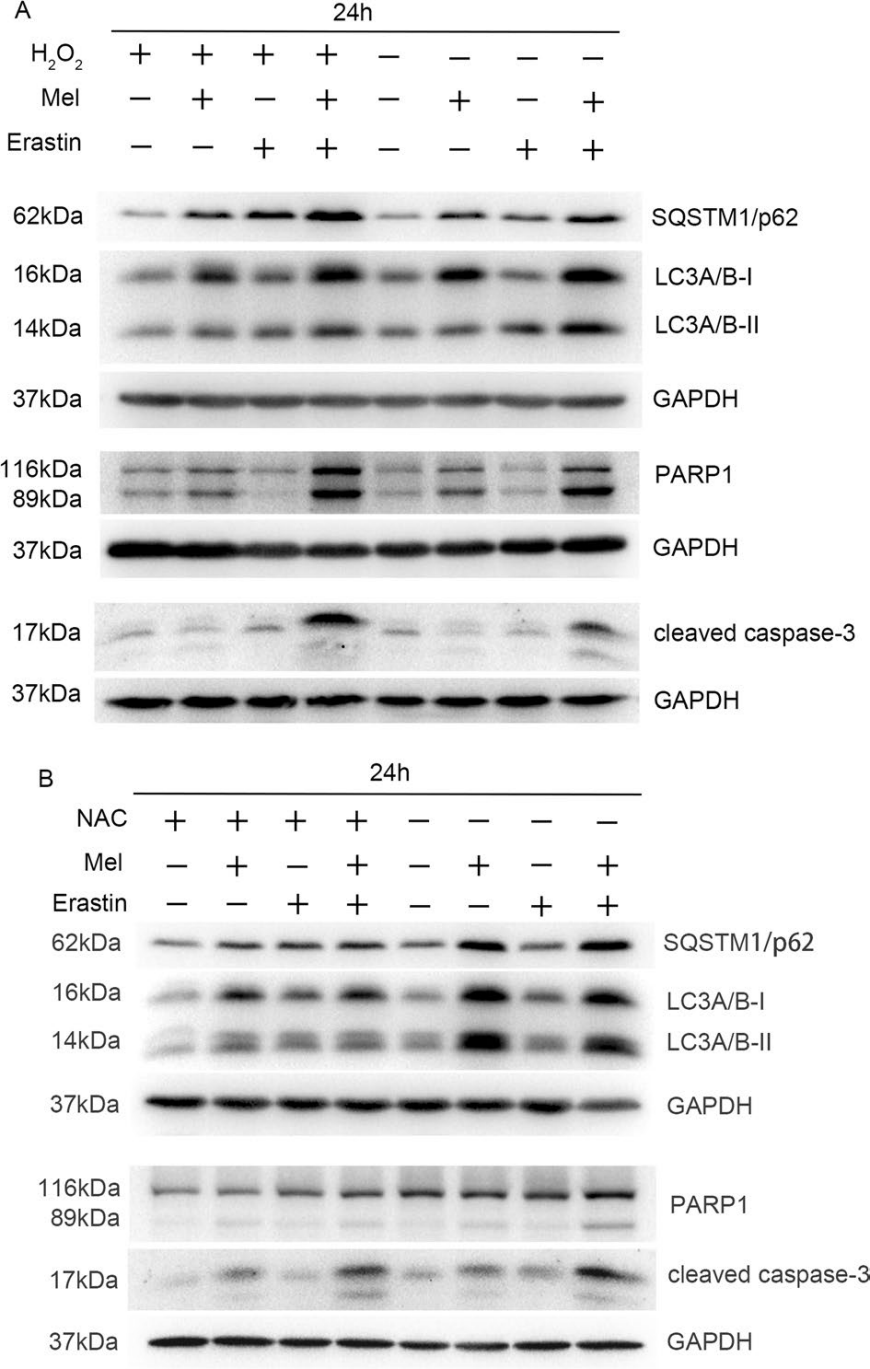
Effects of melatonin (mel) and erastin on autophagy and apoptosis of SCC-15 cells under the treatment of H_2_O_2_ (ROS activator) or NAC (ROS inhibitor). SCC-15 cells were treated with or without 20 μM H_2_O_2_ for 2 h (**A**) or 10 μM NAC for 2 h (**B**) and then 5 μM erastin, 2 mM melatonin, and their combination were added for 24 h. Western blot analysis of the protein expression levels of SQSTM1/p62, LC3A/B, cleaved caspase-3, and PARP1 in SCC-15 cells was conducted. *ns* not significant, *p < 0.05, **p < 0.01, ***p < 0.001

To test whether ROS led to the programmed cell death mentioned above, 20 μM ROS activator (H_2_O_2_) and 10 mM ROS inhibitor [N-acetyl cysteine (NAC)] were added to SCC-15 cells 2 h before treatment with melatonin, erastin, or their combination. The results showed that after the supplement of H_2_O_2_, the expression level of SQSTM1/p62, LC3A/B-II, PARP1, and cleaved-caspase 3 were all increased compared with the corresponding group. Notably, the melatonin + erastin group expressed the highest levels of these proteins among the four groups with H_2_O_2_. In addition, the group with NAC supplement showed decreased level of SQSTM1/p62, LC3A/B-II, PARP1, and cleaved-caspase 3 expression levels, with reference to the corresponding group. Among the four groups, the melatonin + erastin group exhibited the lowest levels of SQSTM1/p62, LC3A/B-II, PARP1, and cleaved-caspase 3. Thus, melatonin- and erastin-induced inhibition of autophagy, and enhanced apoptosis (Fig. 4) and ferroptosis (Additional file 1: Figure S2A–F), were further upregulated by H_2_O_2_ and downregulated by NAC.

### Melatonin combined with erastin enhanced antitumor effects without severe systemic toxicity and side effects in vivo

**Fig. 5:**
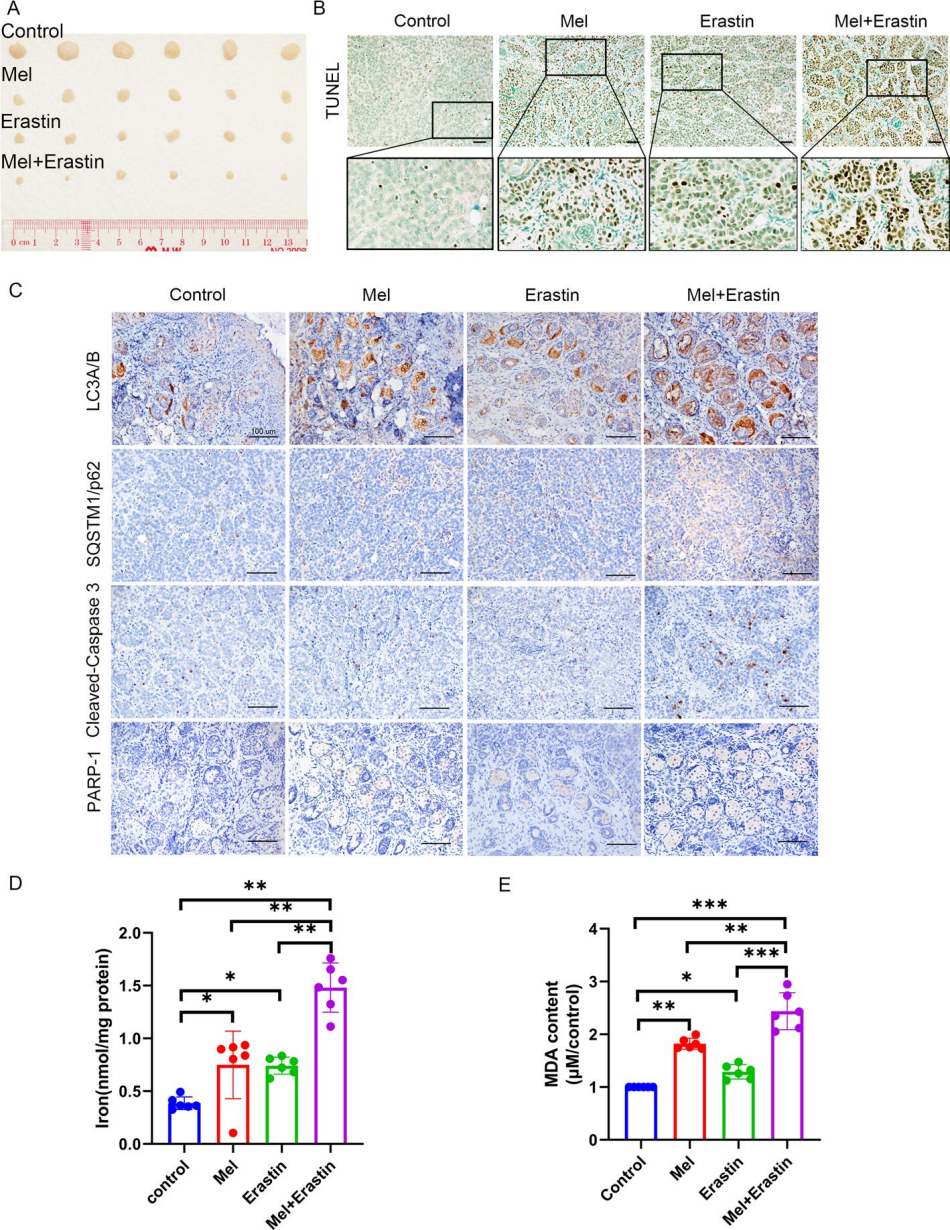
Effects of melatonin and erastin on cell apoptosis, autophagy, and ferroptosis in vivo. **A** Representative photographs of isolated tumor tissues at day 15 after treatment. The tumor tissues from four rows were taken from control group, melatonin group, erastin group, and melatonin + erastin group, respectively. TUNEL levels (**B**), SQSTM1/p62, LC3A/B, cleaved caspase-3, and PARP1 expression levels (**C**) were tested in the isolated tumors at day 15 after treatment. Iron levels in mice serum (**D**) and MDA levels in the isolated tumor tissues (**E**) were measured at day 15 after treatment. *p < 0.05, **p < 0.01, ***p < 0.001. Scale bars: 100 nm

To verify the inhibitory effect of melatonin and erastin on the growth of oral cancer in vivo, a mouse-based subcutaneous oral cancer xenograft model was applied using SCC-15 cells (Additional file 1: Figure S3A–D). After sacrificing the mice, the tumors were isolated (Fig. 5A) and tumor volume (Additional file 1: Figure S3E) and weight (Additional file 1: Figure S3F) were compared among the four groups. Tumor proliferation was considerably inhibited in mice that received intraperitoneal injection of the drugs. Both the melatonin- and erastin-treated groups showed higher anticancer effects than those of the control group (*p* < 0.05), and the melatonin + erastin-treated group exhibited the most obvious inhibition of tumor growth. Moreover, the difference between the melatonin- and erastin-treated groups was not statistically significant (*p* > 0.05). In addition, no notable body weight loss was observed in any of all the four groups (Additional file 1: Figure S3G).

The lungs, liver, spleen, and kidneys were harvested after sacrificing the mice and were embedded in paraffin and stained with hematoxylin and eosin (H&E) (Additional file 1: Figure S4). As expected, no nephrotoxicity, hepatotoxicity, or pulmonary toxicity were observed in any of the four groups, indicating that melatonin and erastin had no severe toxicity or side effects on organs.

### In vivo autophagy inhibition and apoptosis and ferroptosis enhancement are involved in the anticancer effect of combined melatonin and erastin treatment

To further verify the underlying mechanisms by which melatonin and erastin exert anticancer effects in vivo, cell apoptosis and autophagy were evaluated. The TUNEL assay (Fig. 5B) showed that the combined treatment induced more cell apoptosis than melatonin or erastin alone, whereas cell apoptosis was rarely observed in the control group. The IHC results (Fig. 5C) revealed that melatonin combined with erastin resulted in more cell autophagy and less cell apoptosis than other treatments. These data confirmed that the combination of melatonin and erastin effectively inhibited tumor cell autophagy and promoted apoptosis in vivo.

To investigate the tumor cell ferroptosis level in the subcutaneous tumor mouse model, mouse serum iron and MDA levels were detected. The melatonin + erastin-treated group contained the highest levels of Fe^2+^ (Fig. 5D) and MDA (Fig. 5E) and exhibited the highest lipid peroxidation level.

## Discussion

Genetic alterations and abnormal proliferation in cancer cells are associated with high levels of ROS-related oxidative stress. Thus, agents modulating ROS metabolism are able to regulate tumor cell growth [24]. Melatonin, an ROS scavenger [25], exhibits beneficial effects on various pathophysiological conditions characterized by ROS overproduction [26]. However, melatonin also presents prooxidant ability and induces high ROS at high levels [27–29]. In the present study, we demonstrated that low concentration (μM levels) of melatonin could downregulate ROS while high concentrations (mM levels) of melatonin had the opposite effect. The underlying mechanism remains unclear, yet calmodulin activation or mitochondrial dysfunction may be involved [30]. Regulating autophagy and apoptosis under different stressful conditions through the modulation of ROS is a key process in the balance between cell survival and death. Here we proved that melatonin exerted anticancer effects through the regulation of ROS, while more studies are needed to illustrate the crosstalk between ROS and these different cell death processes.

In response to different challenges, such as oxidative stress, multiple forms of cell death may occur. The loss of control over one of multiple types of cell death leads to the development of diseases, including cancer. Therefore, novel drugs controlling the RCD pathway hold great promise for the prevention and treatment of cancers. During the past few decades, many different types of RCD, including apoptosis-, ferroptosis- and autophagy-dependent cell death, have been identified. Nevertheless, more investigations on RCD are needed to illustrate the interactions among distinct cell death signaling pathways.

Most conventional chemotherapeutics (e.g., cisplatin) function by inducing cancer cell apoptosis [31]. Melatonin, as a pleiotropic hormone, has been identified to exert antiapoptosis effects, which seems contradictory to its known anticancer property. Recently, several studies have demonstrated that the modulatory effect of melatonin on apoptosis is cell-type dependent, meaning that melatonin displays a proapoptotic role in cancer cells but an antiapoptotic role in normal cells [32]. The possible mechanism may be related to endoplasmic reticulum stress [33], but further investigations are needed. Moreover, erastin is defined as an inducer of ferropotosis that does not cause apoptosis [34]. Researchers have found that erastin-treated cells did not display apoptosis-related changes in nuclear morphology and did not cause PARP1 and pro-caspase-3 cleavage, while several other studies showed that erastin induced cell apoptosis [35, 36]. The results of flow cytometry and comet assays in the current study proved that erastin increased DNA fragmentation in SCC-15 cells and demonstrated no significant difference in PARP1 and cleaved caspase-3 expression levels, compared with the control group, which was in accordance with a previous study [11]. Moreover, compared to the single usage of melatonin, the combined use of melatonin and erastin upregulated PARP1 and cleaved caspase-3 expression. Herein, erastin failed to induce apoptosis alone, but could promote the effects of melatonin on the apoptosis of SCC-15 cells.

Autophagy has been shown to exert conflicting effects in cancer treatment, as both the stimulation and inhibition of autophagy have been proposed as beneficial for anticancer treatment [37]. These conflicting findings have caused controversy in cancer treatment when targeting autophagy. Therefore, it is highly warranted to illuminate the mechanisms underlying the uncertain effects of autophagy on oral cancers. The regulatory effects of melatonin on autophagy are inconclusive as well [38]. In this study, both SQSTM1/p62 and LC3A/B were upregulated in SCC-15 cells following treatment with melatonin and erastin. The upregulation of LC3A/B and the consumption of SQSTM1/p62 represent the fluent process of autophagy signaling pathway, thus, our results indicated that melatonin and erastin may trigger the initiation of autophagy, which is blocked downstream.

Early studies suggested that ferroptosis is morphologically, biochemically, and genetically distinct from autophagy. Conversely, studies in recent years have demonstrated that the activation of ferroptosis is dependent on the induction of autophagy, and many ferroptosis regulators such as SLC7A11, GPX4, NRF2, p53, and ACSL4 have been identified as potential regulators of autophagy [39, 40]. Moreover, autophagy-dependent ferroptosis also exhibits tumor heterogeneity, which means different biological behaviors and dynamic characteristics of cell death [41]. In the present study, we found that the combined use of melatonin and erastin can significantly augment the expression of both SQSTM1/p62 and LC3A/B and enhance the cellular iron level. Thus, the combined use of melatonin and erastin may initiate autophagy, leading to ferroptosis in cancer cells.

Since ROS play an essential role in RCD, and our results showed that the cellular ROS level was upregulated by the combination of melatonin and erastin, it is reasonable to speculate that ROS might be a key regulator in this process. Notably, the melatonin- and/or erastin-induced inhibition of autophagy and enhanced apoptosis and ferroptosis were further upregulated by ROS activator and downregulated by ROS inhibitor. Thus, melatonin and erastin may modulate RCD through the regulation of ROS in cancer cells, while the detailed mechanism remains unclear. It has been demonstrated that Keap1/Nrf2 pathway might be involved in the interaction between autophagy and antioxidant response [42], as well as the regulation effects of ROS on ferroptosis [43] and apoptosis [44]. Therefore, more possible mechanisms underlying the modulation effects of melatonin and erastin on RCD are worth being investigated.

## Conclusions

The combined use of melatonin and erastin exhibits synergistic antioral cancer effects by activating different types of RCD, including apoptosis, autophagy, and ferroptosis, through the modulation of ROS. Novel strategies targeting the switch between different forms of RCD could improve anticancer efficacy and overcome drug resistance, which might be a promising approach to fight against oral cancer and other malignant tumors.

## Supplementary Information

**Additional file 1**. Supplementary figures.

## Data Availability

All data presented in this study are included within the paper and its additional files.

## Abbreviations

ROS: Reactive oxygen species
RCD: Regulated cell death
OSCCs: Oral squamous cell carcinomas
GSH: Glutathione
IHC: Immunohistochemistry
SDS‒PAGE: Sodium dodecyl sulfate‒polyacrylamide gel electrophoresis
TBARS: Thiobarbituric acid reactive substances
NAC: N-acetyl cysteine
H&E: Hematoxylin and eosin
