# Supplementary material for: Melatonin and erastin emerge synergistic anti-tumor effects on oral squamous cell carcinoma by inducing apoptosis, ferroptosis, and inhibiting autophagy through promoting ROS
Source: Cell Mol Biol Lett. 2023 May 2;28:36. doi: 10.1186/s11658-023-00449-6 (PMC10155313; doi:10.1186/s11658-023-00449-6)
Supplement: Supplementary file 1 — Additional file 1. Supplementary figures. [file 11658_2023_449_MOESM1_ESM.docx]

**Supplementary Information**

**Synergistic effect of melatonin and erastin may target apoptosis and DNA damage and repair signaling pathway**

To find the possible pathway involved in the synergistic anticancer effect of melatonin and erastin, RT^2^ Profiler PCR Array (Cancer Pathway Finder) was used to detect the relevant changes in gene expression among the four groups: control group, melatonin-treated group, erastin-treated group and melatonin + erastin-treated group. The BIRC3, caspase 2, caspase 9, DDIT3, GADD45G, POLB and PPP1R15A expression levels were increased in the treatment groups, with reference to the control group. Notably, the melatonin + erastin-treated group exhibited the highest expression level among all groups. In conclusion, the signaling was enriched in apoptosis and DNA damage and repair. To verify the mRNA and protein expression levels of these genes, qRT-PCR and western blot were performed. However, no significant difference was found among the four groups.

**Supplementary Figure 1.** PCR array of Human Cancer Pathway Finder genes in SCC-15 cells. SCC-15 cells were treated with 5 μM erastin, 2 mM melatonin and their combination for 24 h. PCR array were conducted. Heat-map of PCR array results (A). Relative mRNA expressions of the changed genes were showed in (B).

**Supplementary Figure 2.** Effects of H_2_O_2_ (ROS activator) and NAC (ROS inhibitor) on ferroptosis of SCC-15 cells. SCC-15 cells were treated with melatonin (0.5 mM), erastin (0.5 μM), melatonin (2 mM), erastin (5 μM) and their combination for 24 h with/without 20 μM H_2_O_2_ or 10 mM NAC for 2 h. The expression of cellular ROS concentration (A), MDA content (B), relative lipid ROS (C), GSH concentration (D) iron concentration (E) and glutamate concentration (F) were tested (* *p* < 0.05, ** *p* < 0.01, *** *p* < 0.001).

**Supplementary Figure 3.** Effects of melatonin (Mel) and erastin on mouse-based subcutaneous oral cancer xenograft models. Representative photographs of established mouse-based subcutaneous oral cancer xenograft models at day 15 after treatment are shown (A-D). Mice were treated with DMSO (control group) (A), melatonin (100 mg/kg/i.p., every 2 days) (B), erastin (30 mg/kg/i.p., every 2 days) (C), and melatonin (100 mg/kg/i.p., every 2 days) + erastin (30 mg/kg/i.p., every 2 days) (D) for seven cycles. Tumor volume was assessed every 2 days (E). Tumor weight of isolated tumor tissues at day 15 after treatment (F). Mice weights were measured every 2 days (G). Experiments were repeated three times with at least 6 mice, and the data are expressed as the mean ± SEM. (* *p* < 0.05, ** *p* < 0.01, *** *p* < 0.001).

**Supplementary Figure 4.** Effects of melatonin and erastin on the systemic conditions of the mouse-based subcutaneous oral cancer xenograft models. Hematoxylin-eosin staining of Lung, liver, kidney and spleen isolated from mice at day 15 after treatment (A). Scale bars: 100 nm.
